# Estimating How Sounds Modulate Orientation Representation in the Primary Visual Cortex Using Shallow Neural Networks

**DOI:** 10.3389/fnsys.2022.869705

**Published:** 2022-05-09

**Authors:** John P. McClure, O. Batuhan Erkat, Julien Corbo, Pierre-Olivier Polack

**Affiliations:** ^1^Center for Molecular and Behavioral Neuroscience, Rutgers University–Newark, Newark, NJ, United States; ^2^Behavioral and Neural Sciences Graduate Program, Rutgers University–Newark, Newark, NJ, United States

**Keywords:** neuronal representations, shallow neural network, primary visual cortex (V1), sound modulation, orientation representation, audiovisual detection task, sensory processing

## Abstract

Audiovisual perception results from the interaction between visual and auditory processing. Hence, presenting auditory and visual inputs simultaneously usually improves the accuracy of the unimodal percepts, but can also lead to audiovisual illusions. Cross-talks between visual and auditory inputs during sensory processing were recently shown to occur as early as in the primary visual cortex (V1). In a previous study, we demonstrated that sounds improve the representation of the orientation of visual stimuli in the naïve mouse V1 by promoting the recruitment of neurons better tuned to the orientation and direction of the visual stimulus. However, we did not test if this type of modulation was still present when the auditory and visual stimuli were both behaviorally relevant. To determine the effect of sounds on active visual processing, we performed calcium imaging in V1 while mice were performing an audiovisual task. We then compared the representations of the task stimuli orientations in the unimodal visual and audiovisual context using shallow neural networks (SNNs). SNNs were chosen because of the biological plausibility of their computational structure and the possibility of identifying *post hoc* the biological neurons having the strongest influence on the classification decision. We first showed that SNNs can categorize the activity of V1 neurons evoked by drifting gratings of 12 different orientations. Then, we demonstrated using the connection weight approach that SNN training assigns the largest computational weight to the V1 neurons having the best orientation and direction selectivity. Finally, we showed that it is possible to use SNNs to determine how V1 neurons represent the orientations of stimuli that do not belong to the set of orientations used for SNN training. Once the SNN approach was established, we replicated the previous finding that sounds improve orientation representation in the V1 of naïve mice. Then, we showed that, in mice performing an audiovisual detection task, task tones improve the representation of the visual cues associated with the reward while deteriorating the representation of non-rewarded cues. Altogether, our results suggest that the direction of sound modulation in V1 depends on the behavioral relevance of the visual cue.

## Introduction

Multi-sensory integration leads to a multimodal unified percept. It was long thought that multimodal integration was performed in higher-order multisensory cortices such as the posterior parietal cortex (Molholm et al., 2006; Song et al., 2017) once the parallel unimodal processing of the different sensory modalities was completed. However, several recent studies have demonstrated the presence of direct mutual anatomical connections (Falchier et al., 2002; Rockland and Ojima, 2003; Cappe and Barone, 2005; Iurilli et al., 2012; Ibrahim et al., 2016; Deneux et al., 2019; Garner and Keller, 2021) and cross-modal sensory processing modulations (Iurilli et al., 2012; Ibrahim et al., 2016; Meijer et al., 2017; Deneux et al., 2019; Knöpfel et al., 2019; McClure and Polack, 2019; Garner and Keller, 2021) at the earliest stages of cortical sensory processing in primates and rodents. Hence, we have recently demonstrated that sounds modulate the visually evoked response of neurons of the primary visual cortex (V1) to the presentation of oriented stimuli (McClure and Polack, 2019). We showed that sounds potentiate the responses of neurons well-tuned to the stimulus orientation and direction while suppressing the responses of neurons not tuned for the orientation and/or the direction of the visual cue. As a result, sound modulation improved the representation of the orientation and the direction of the visual stimulus in V1 (McClure and Polack, 2019). If studies on cross modal interactions have mainly reported facilitatory interactions (Vroomen and De Gelder, 2000; Odgaard et al., 2004; Lippert et al., 2007; Gleiss and Kayser, 2014), others have shown context-dependent suppressive effects (Iurilli et al., 2012; Hidaka and Ide, 2015; Meijer et al., 2017; Deneux et al., 2019). Altogether, those studies suggest that the sign of sound modulation in V1 depends on the behavioral relevance of the visual and auditory stimuli.

To test this hypothesis, we performed calcium imaging in the V1 of mice alternating, during the same recording session, between the performance of a unimodal visual and an audiovisual task. To compare the representations of the visual stimuli in V1 between the unimodal and the audiovisual context, we tested a novel approach for this type of analysis: the shallow neural networks (SNNs). SNNs are simple neural networks having only one or two inner layers. Like other neural networks, they are classifiers that can be trained at identifying patterns with a very high proficiency (Fukushima, 1980). This approach was selected to fulfill the following criteria: (1) to be as biologically plausible as possible; (2) to use all the recorded neurons as an input instead of requiring the selection of “active” neurons; (3) to be able to classify V1 responses to orientations that do not belong to the training set; and (4) to allow determining which neurons carry the most weight in the classification of the visual stimulus. SNNs fulfill those four criteria as: their structure is inspired by the computational structure of the visual cortex (Fukushima, 1980); they do not require any criteria-based selection from the experimenter for including neurons; their output being a vector of probabilities assigned to each orientation of the training set, we can take advantage of the continuity of the orientation space and use circular statistics to decode any orientation; finally, their simplicity (i.e., their shallowness) allows for a straightforward access to the weight given in the classifying decisions to each individual recorded neuron.

We first tested the SNN approach using a calcium imaging dataset from a prior study to investigate how pure tones affect the representation of oriented stimuli in the V1 L2/3 of mice passively receiving the stimuli (McClure and Polack, 2019). We found that the weight assigned to each recorded neuron by the classifier during training was highly correlated with the neuron’s tuning properties (preferred orientation and selectivity), suggesting that an optimal classifier uses the same features of the neuronal responses that we capture with the traditional approach of orientation tuning curves. Then, we used the trained SNNs to classify orientations that were not part of the training set. We showed that the presentation of a pure tone improved the visual stimulus representation. Those results reproduced the findings obtain when analyzing the same database using an active neurons selection approach (McClure and Polack, 2019). Then, we extended the method to our new dataset and showed that when pure tones have a behavioral relevance in the audiovisual task, the modulation of the representation of visual information in V1 can be bidirectional.

## Materials and Methods

All the procedures described below have been approved by the Institutional Animal Care and Use Committee (IACUC) of Rutgers University–Newark, in agreement with the Guide for the Care and Use of Laboratory Animals (National Research Council of the National Academies, 2011).

### Surgery

#### Head-Bar Implants

Ten minutes after systemic injection of an analgesic (carprofen, Zoetis, Parsippany-Troy Hills, NJ, United States; 5 mg per kg of body weight), adult (3–6 months old) male and female Gad2-IRES-Cre (Jackson stock #019022) × Ai9 (Jackson stock #007909) mice were anesthetized with isoflurane (5% induction, 1.2% maintenance) and placed in a stereotaxic frame. Body temperature was kept at 37°C using a feedback-controlled heating pad. Pressure points and incision sites were injected with lidocaine (2%). Eyes were protected from desiccation with artificial tear ointment (Dechra, Northwich, United Kingdom). Next, the skin covering the skull was incised and a custom-made lightweight metal head-bar was glued to the skull using Vetbond (3M, Saint Paul, MN, United States). In addition, a large recording chamber capable of retaining the water necessary for using a water-immersion objective was built using dental cement (Ortho-Jet, Lang, Dental, Wheeling, IL, United States). Mice recovered from surgery for 5 days, during which amoxicillin was administered in drinking water (0.25 mg/mL).

#### Adeno-Associated Virus (AAV) Injection

After recovery from the head-bar surgery, mice were anesthetized using isoflurane as described above. A circular craniotomy (diameter = 3 mm) was performed above V1. The AAV vector AAV1.eSyn.GCaMP6f.WPRE.SV40 (UPenn Vector Core, Philadelphia, PA, United States) carrying the gene of the fluorescent calcium sensor GCaMP6f was injected at three sites 500 μm apart around the center of V1 (stereotaxic coordinates: −4.0 mm AP, +2.2 mm ML from bregma) using a MicroSyringe Pump Controller Micro 4 (World Precision Instruments, Sarasota, FL, United States) at a rate of 30 nl/min. Injections started at a depth of 550 μm below the pial surface and the tip of the pipette was raised in steps of 100 μm during the injection, up to a depth of 200 μm below the dura surface. The total volume injected across all depths was 0.7 μl. After removal of the injection pipette, a 3-mm-diameter coverslip was placed over the dura, such that the coverslip fits entirely in the craniotomy and was flush with the skull surface. The coverslip was kept in place using Vetbond and dental cement. Mice were left to recover from the surgery for at least 3 weeks to obtain a satisfactory gene expression.

### Functional Imaging

#### Calcium Imaging Setup

During the last week of recovery, mice were trained to stay on a spherical treadmill consisting of a ball floating on a small cushion of air that allowed for full 2D movement (Polack et al., 2013). During three daily 20-min sessions, the mouse head-bar was fixed to a post holding the mouse on the apex of the spherical treadmill. Ball motion was tracked by an IR camera taking pictures of the ball at 30 Hz. Eye motion was monitored at 15 Hz using a second IR camera imaging the reflection of the eye on an infrared dichroic mirror. Functional imaging was performed at 15 frames per second using a resonant scanning two-photon microscope (Neurolabware, West Hollywood, CA, United States) powered by a Ti-Sapphire Ultra-2 laser (Coherent, Santa Clara, CA, United States) set at 910 nm. The microscope scanning mirrors were hermetically sealed in a chamber to bring the scanning hum below the room ambient noise (<59 dBA). The laser beam was focused 200 microns below the cortical surface using a 16×, 0.8 NA Nikon water-immersion objective. The objective was tilted 30° such that the objective lens was parallel to the dura surface. Laser power was kept below 70 mW. Frames (512 × 796 pixels) were acquired using the software Scanbox developed by Neurolabware.

#### Naïve Imaging Session

Mice were placed head fixed in the functional imaging rig in front of a screen such that it covered the visual field of the right eye, contralateral to the craniotomy. Visual stimuli of the test block consisted of the presentation of one of two vertical sinewave gratings that drifted toward the right and were rotated clockwise by 45° and 135° (temporal frequency = 2 Hz, spatial frequency = 0.04 cycle per degree, contrast = 75%; duration: 3 s; intertrial interval: 3 s). Visual cues were presented in a pseudorandom order, such as the same stimulus could not be presented more than three times in a row. At the end of the imaging session, after a break of at least 5 min, we assessed the orientation tuning of the imaged neurons by presenting an orientation tuning block that consisted of the presentation of a series of drifting sinewave gratings (12 orientations evenly spaced by 30° and randomly permuted). The spatiotemporal parameters of the orientation tuning stimuli were identical to those for the task block except for their duration (temporal frequency = 2 Hz, spatial frequency = 0.04 cycle per degree, contrast = 75%; duration: 1.5 s; intertrial interval: 3 s). Auditory stimuli consisted of the presentation of one of two sine wave pure tones (10 kHz and 5 kHz; 78 dB; duration: 3 s). Each audiovisual trial resulted from the random combination of one of the two pure tones with one of the two drifting gratings (four possibilities: 5 kHz tone + 45° drifting grating, 10 kHz tone + 45° drifting grating, 5 kHz tone + 135° drifting grating, and 10 kHz tone + 135° drifting grating). As scanning was not synced to the stimuli, a photodiode located at the top left corner of the screen was used to detect the exact timing of the visual stimulus onset and offset. The photodiode signal was acquired along with the following signals: (1) a signal provided by the two-photon microscope, which indicated the onset of each frame, and (2) two analog signals encoding the orientation of the drifting grating. These signals were digitized (NiDAQ, National Instruments, Austin, TX, United States) and recorded with the software WinEDR (John Dempster, University of Strathclyde). Imaging sessions started by recording one thousand frames with the green and red channels. The red channel was used to exclude GABAergic neurons from the analysis.

#### Behavioral Training

After the naïve recording session, mice were water-deprived up to 85% of their body weight and acclimated to head fixation on a spherical treadmill in custom-built, soundproof training rigs. Each rig was equipped with a monitor (Dell), a water dispenser with a built-in lickometer (to monitor licking, infrared beam break). Data acquisition boards (National Instruments and Arduino) were used to actuate water delivery and vacuum reward retrieval as well as monitor animal licking. The monitor and data acquisition boards were connected to a computer that ran the custom-made training program scripted in MATLAB (MathWorks, Natick, MA, United States). Once animals reached the target weight and were acclimated to the training setup, they were trained to perform the orientation discrimination task. In this task, drifting sine-wave gratings oriented 45° below the vertical were paired with a water reward, and the animal was expected to lick (Go). Drifting gratings orthogonal to the Go stimulus signaled the absence of reward, and the animal was expected to withhold licking (NoGo, orientation 135°) during those trials. When the stimulus instructed the animal to lick, the water delivery had to be triggered by the mouse licking during the third second of the stimulus presentation. No water was dispensed in the no-lick condition or if the mouse failed to trigger water delivery in the lick condition. If the animal responded correctly [Hit or Correct Rejection (CR)], the intertrial interval was 3 s. If the animal responded incorrectly [Miss or False Alarm (FA)], the intertrial interval was increased to 9.5 s as negative reinforcement. Animals were considered experts if their performance during training sessions was greater than 1.7 (probability of chance behavior <0.1%, Monte Carlo simulation; Einstein et al., 2017). All the mice were also trained to perform the same task but using the 5 kHz tone as a NoGo cue and the 10 kHz tone as the Go cue. Half of the mice started training with the visual task while the other half started training with the auditory task. The order of training did not impact the mice’s performance.

#### Recording Sessions

As training was performed on a training setup located in a different room, trained mice were habituated to perform the task on the imaging setup (typically for one or two sessions) until they could perform the task above the expert threshold. Recording sessions consisted of five blocks (see Figure 5D). The first block contained unimodal (either visual or auditory; the modality was selected at random at the beginning of the session) and was followed by an audiovisual block. For trained mice, the reward in this audiovisual block was associated to the modality (visual or auditory) of the preceding unimodal block. Hence if the first block was unimodal visual, the second block was audiovisual with the visual cue indicating the presence or absence of reward. The third block was a unimodal block (visual if the first unimodal block was auditory, auditory if the first unimodal block was visual). The fourth block was an audiovisual block (same rule for the reward as for the second block). The last block was an orientation tuning block, consisting in the presentation in pseudorandom order of twelve evenly spaced oriented visual stimuli (0°, 30°, 60°, 90°, 120°, 150°, 180°, 210°, 240°, 270°, 300°, 330°; the 12 orientations needed to be presented before starting a new series).

### Data Analysis

All the analyses detailed below were performed using custom MATLAB scripts.

#### Imaging Data Pre-processing

Calcium imaging frames were realigned offline to remove movement artifacts using the Scanbox algorithm (Neurolabware). A region of interest (ROI) was determined for each neuron using a semi-automatic segmentation routine. For every frame, the fluorescence level was averaged across the pixels of the ROI. Potential contamination of the soma fluorescence by the local neuropil was removed by subtracting the mean fluorescence of a 2–5 μm ring surrounding the neuron’s ROI, excluding the soma of neighboring neurons, and then adding the median value across time of the subtracted background. We then computed the fractional fluorescence from the background-subtracted fluorescence data. The fractional fluorescence (Δ*F*/*F* = (*F*–*F*_0_)/*F*_0_), was calculated with *F*_0_ defined as the median of the raw fluorescence measured during every inter-trial interval. The response of neurons to a trial was then measured as the mean fractional fluorescence measured during the first second of the visual stimulus presentation minus the mean fractional fluorescence measured during the second and a half preceding the stimulus presentation. The orientation tuning curve of each neuron was computed using a resampling-based Bayesian method (Cronin et al., 2010; McClure and Polack, 2019) from the area under the curve of the fractional fluorescence responses recorded during the different trials of the tuning curve blocks. The preferred orientation was defined as the peak of the orientation tuning curve. When a neuron was not direction selective (i.e., responding equally to the same oriented stimulus moving in opposite directions), the preferred orientation was defined as the orientation included in the range (0°–180°). The responses of all the neurons to all the trials as well as the neurons’ tuning curve parameters were stored in a SQL database.

### Shallow Neural Networks

#### Rationale for the Choice of Shallow Neural Networks

The SNN approach was selected to be as biologically plausible as possible (criterium #1). Indeed, the structure of neural networks was inspired by the computational structure of the visual cortex (Fukushima, 1980), and it was shown that neural networks provide pertinent computational models of the visual cortex (Lindsay, 2021). Therefore, we can assume that the output of the SNN in this study might have a biological relevance. We also wanted the classifier to work on the entire recorded population and therefore not to require the selection of “active” neurons (criterium #2). Indeed, the activity of cortical neurons follows a long-tailed gamma or log-normal distribution (Decharms and Zador, 2000; Wohrer et al., 2013). As a result, most neurons’ evoked activity is very similar to their resting state and only a few neurons significantly increase their firing rate when a visual stimulus is presented (Barth and Poulet, 2012; Wohrer et al., 2013). Therefore, simple strategies of analysis such as averaging the activity across recorded neurons have limited interpretative power, as they are poorly sensitive to the change of activity of the minority of neurons responding to the stimulus. A common strategy used to circumvent this issue is to determine a threshold above which neurons are considered “active.” However, this approach reveals limitations when working on the modulation by the behavioral context of the V1 population activity. Indeed, those extrinsic modulatory factors inactivate some neurons while activating others (Iurilli et al., 2012; Ibrahim et al., 2016; Meijer et al., 2017; McClure and Polack, 2019), complicating the comparison of the different “active” populations responding in the different behavioral contexts. To investigate sensory representations, some other analysis strategies such as dimensionality reduction (Cunningham and Yu, 2014; Carrillo-Reid et al., 2019) or decoding (Quian Quiroga and Panzeri, 2009; Stringer et al., 2021) avoid selecting neurons. These approaches capture the availability of the information about the stimulus feature embedded in the population activity using diverse metrics of statistical distance between different arrays of data. However, because the result of those computations is abstract, those strategies can only provide limited insights about the implementation by the biological networks of the computations realized by those methods. Indeed, if those techniques inform us about *what* information is present and can point out discrepancies between what an optimal decoder and an animal can discriminate (Stringer et al., 2021), they do not directly tackle the question of *how* the available information is used by the biological system. This is not the case of the SNN whose output allows taking advantage of the continuity of the orientation space to determine the representation in V1 of visual stimuli that do not belong to the classifier training stimuli. Finally, the simplicity of SNNs (i.e., their shallowness) allows to straightforwardly access the weights assigned to each recorded neuron in the classifying decisions. Here, we chose the *Connection Weight Approach* (Olden and Jackson, 2002; Olden et al., 2004) to determine which neurons carry the most weight in the classification of the visual stimulus.

#### Implementation

The shallow neural network was a two-layer feedforward network with a sigmoid transfer function in the hidden layer and a softmax transfer function in the output. It was generated in MATLAB using the *patternnet* function. The input layer was made of 250 *computational neurons* that receive the evoked activity of 250 *cortical neurons*, the single hidden layer was made of 10 hidden computational neurons, and the output layer was composed of 12 computational neurons corresponding to the 12 orientations of the tuning block. The input layer was connected to 250 cortical neurons randomly selected in either the naïve or trained mouse database using an SQL query. This sampling method pools cortical neurons from different mice. Pooling neurons across mice breaks the correlational structures between neurons. However, those correlations were found to have little influence on sensory information encoding. Indeed, although correlations are prevalent in the visual cortex, the additional information they provide is small (about 4% in an analysis window greater than 100 ms; Golledge et al., 2003) and offset by the redundancy arising from the neurons’ similar tuning properties (Montani et al., 2007). As a result, the spaces encoding sensory and behavioral variables are essentially orthogonal (Stringer et al., 2019; Rumyantsev et al., 2020). As the cortical neurons used for each SNN were selected from different recording sessions, the SNNs were trained using resampled trials from the tuning block (100 resampled trials for each of the 12 orientations). Those resampled trials consisted in the random selection for each selected cortical neuron of one trial corresponding to the presentation of that stimulus. The network was trained by scaled conjugate gradient backpropagation using the *trainscg* MATLAB function. For the SNN training, the data was split into training, validation, and test sets: 70% for training; 15% for cross-validation to validate that the network is generalizing and to stop training before overfitting, and 15% to independently test network generalization.

#### Cortical Neuron’s Connection Weights

We evaluated the relative weight of the cortical neurons connected to each input computational neuron using the *Connection Weight Approach* (Olden and Jackson, 2002; Olden et al., 2004). First, input-hidden-output connection weights were obtained as the product of input-hidden and hidden-output connection weights for each input and hidden computational neuron; then overall connection weights were defined as the sum of the input-hidden-output connection weights for each input variable (Olden and Jackson, 2002). This approach that uses raw input-hidden and hidden-output connection weights in the neural network provides the best methodology for accurately quantifying variable importance (Olden et al., 2004).

### Statistics

#### Permutation Tests

To determine if the mean across trials computed from two different pools was significantly different, we compared the value obtained from the distribution of 1,000 or 10,000 differences obtained when the pool labels were shuffled. The two-tailed confidence intervals of the null hypothesis at the alpha level 0.05 were defined as the 2.5 and 97.5 percentile of the distribution obtained from the permutations. The difference between the observed means was considered significant if located outside the confidence interval of the null distribution.

#### Circular Statistics

Circular statistics were computed with the Circular Statistics Toolbox for MATLAB (Berens, 2009).

## Results

### Representation of the Visual Stimulus Orientation in the Naïve Mouse V1

**FIGURE 1:**
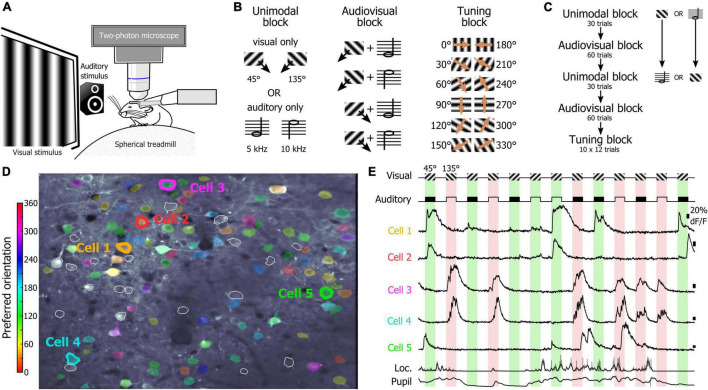
Recording sessions in naïve mice. **(A)** Schematic representation of the recording setup. **(B)** Stimuli presented during the unimodal, audiovisual, and tuning blocks. **(C)** Organization of the unimodal, audiovisual, and tuning blocks in a recording session. **(D)** Two-photon image of V1 neurons recorded in a naïve mouse. The preferred orientation of the segmented neurons is indicated by the color scale in inset. White contours indicate segmented neurons that were not estimated orientation selective by the algorithm. **(E)** Example of the activity of neurons indicated in panel **(D)** during the presentation of the visual stimuli of the audiovisual block. The neuronal activity was recorded simultaneously with the locomotion of the animal as well as the pupil size (bottom traces).

**FIGURE 2:**
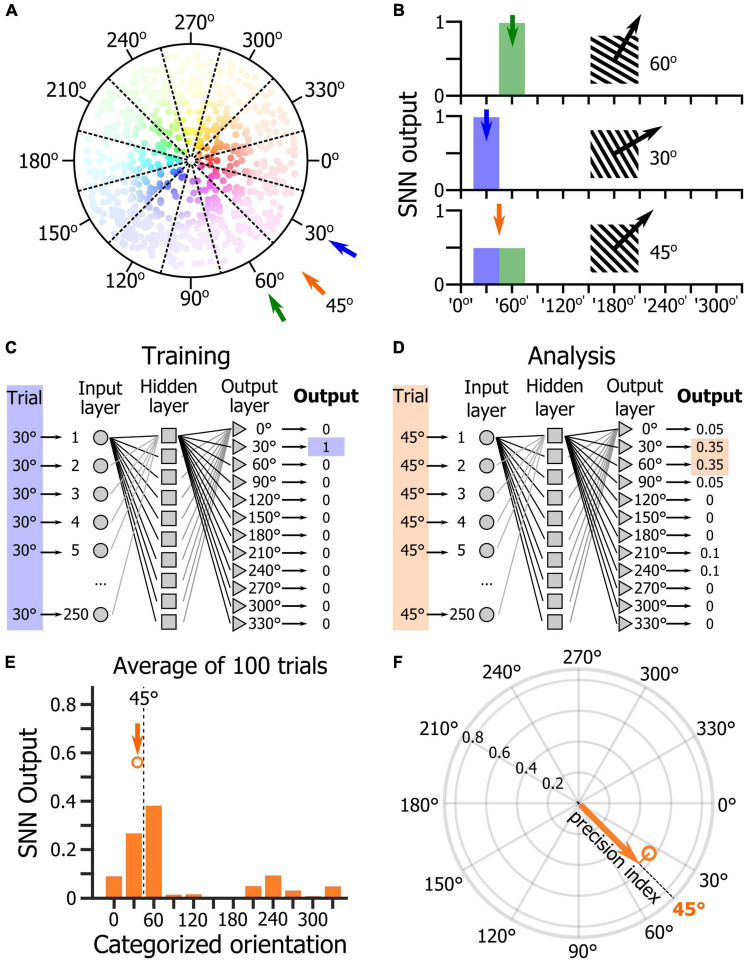
Analysis of orientation representation in V1 using shallow neural networks. **(A)** Schematic representation of the distribution of the preferred orientations of V1 neurons. Each dot represents a neuron. The hue represents the preferred orientation of the neuron, and the hue intensity represents the amplitude of the neuronal response to the presentation of its preferred orientation. **(B)** Schematic representation of the response of a SNN trained to discriminate the V1 population response to the presentation of a 30° drifting grating (top panel) and to the presentation of a 60° drifting grating (middle panel). When the SNN is provided with the response of its input cortical neuron population for the presentation of a 45° drifting grating (bottom panel), its output should indicate an equal probability that the stimulus belongs to the ‘30°’ and ‘60°’ categories. **(C)** Schematic representation of the SNN training. **(D)** Schematic representation of the SNN testing with a 45° stimulus that does not belong to the training categories. **(E)** Average output across 100 trials of the presentation of a 45° drifting grating of a randomly selected trained SNN. The dot indicates the orientation and length of the circular mean vector computed from the mean distribution of the SNN output. **(F)** Precision index defined as the length of the vector resulting from the projection of the circular mean vector onto the axis of the visual stimulus orientation.

One of the main goals of this study was to determine the relevance of using an SNN to assess how the orientations of drifting gratings were represented at the population level by V1 layer 2/3 (L2/3) neurons. To test this approach, we used a dataset of two-photon calcium imaging experiments in which mice placed on a spherical treadmill in front of a screen and a speaker were shown visual, auditory, and audiovisual stimuli (Figure 1A). During the recording sessions, three types of stimulus blocks were presented (Figure 1B): unimodal blocks consisting of either visual or auditory stimuli (45° and 135° drifting gratings or auditory: 5 kHz or 10 kHz sinewave tones, respectively), an audiovisual block (where the two visual and the two auditory cues were randomly paired), and a tuning block (during which series of 12 different drifting gratings were presented). The first block of the session was a unimodal block either visual or auditory, followed by an audiovisual block. Then, the alternate unimodal block was presented (either auditory or visual, respectively) followed by a second audiovisual block (Figure 1C). Each recording session ended with the presentation of a tuning block to allow determining the tuning-curves of the imaged neurons (Figure 1C). Calcium imaging was performed while simultaneously tracking the locomotion and the pupil size (Figures 1D,E) as locomotor activity and arousal (correlated to the pupil size) modulate the neuronal response of V1 neurons (Niell and Stryker, 2010; Polack et al., 2013; Vinck et al., 2015). We had already analyzed this database in a previous study and shown that sound modulation improves the representation of the orientation and direction of the visual stimulus in V1 L2/3 (McClure and Polack, 2019). We had also shown that arousal and locomotion are similar in the unimodal and audiovisual blocks in this dataset (McClure and Polack, 2019). The analytic method applied in that study was a thresholding approach used to determine which neurons were included in the analysis. In this study, we wanted to use a method that allows determining how the V1 population is representing the orientation of the visual stimulus without having to select “responsive neurons” in the recorded neuronal population. We decided to test SNNs as they are very effective for pattern classification (Fukushima, 1980) and therefore were a good candidate to identify the population activity patterns evoked by specific oriented stimuli. For this, we trained single hidden layer SNNs to identify the neuronal patterns evoked by 12 different drifting gratings evenly spaced in the orientation space (note that we will use the term “orientation” to indicate both the orientation and drifting direction of the gratings). The SNN output estimates the probability that the presented pattern belongs to each of the output categories. Hence, a SNN presented with a pattern that it has been trained to identify, theoretically returns an output of 1 for the corresponding category (30° and 60° in the example shown in Figures 2A,B). Because of the continuity of the orientation space, we then assumed that the presentation of an oriented stimulus equidistant from two trained orientations such as 45° (which is equidistant from 30° and 60°) would be classified as 50% “30° drifting grating” and 50% “60° drifting grating” (Figure 2B), as it activates a subpopulation of neurons responding both to the 30° and 60° stimuli (Figure 2A). Hence, we trained SNNs to classify the neural patterns of subpopulations of V1 neurons (250 cortical neurons randomly picked in a database of 1,353 imaged neurons in eight mice). Each SNN was composed of an input layer of 250 computational neurons fully connected to a layer of 10 hidden computational neurons which were in turn fully connected to an output layer of 12 computational neurons (Figure 2C). The training of the network was performed using 100 resampled trials for each of the twelve evenly spaced oriented visual stimuli presented during the tuning block (0°, 30°, 60°, 90°, 120°, 150°, 180°, 210°, 240°, 270°, 300°, 330°). Each resampled trial for an orientation corresponded to the random selection for each cortical neuron of one response to the presentation of that stimulus (mean ΔF/F across the visual stimulus presentation). Once trained, the SNNs were able to accurately classify all the resampled trials (probability of correct classification with cross-validation > 0.99). We then used the trained SNNs to classify 100 resampled trials collected when presenting visual stimuli of the unimodal block, i.e., drifting gratings having orientations that the SNNs were not trained to recognize (45° and 135°; Figure 2D). The 100 outputs of each SNN were averaged and the circular mean of this mean output provided the orientation estimated by the SNN (Figure 2E). The accuracy (precision index) of this represented orientation was measured as the projection of the circular mean vector onto the radial axis of the visual stimulus orientation (Figure 2F). To determine the variability of the representation across the population of imaged V1 neurons, we repeated the analysis hundreds of times, creating each time a new SNN from a new pseudo-population of 250 V1 neurons.

### Relationship Between Tuning Curves and Shallow Neural Network Weights

**FIGURE 3:**
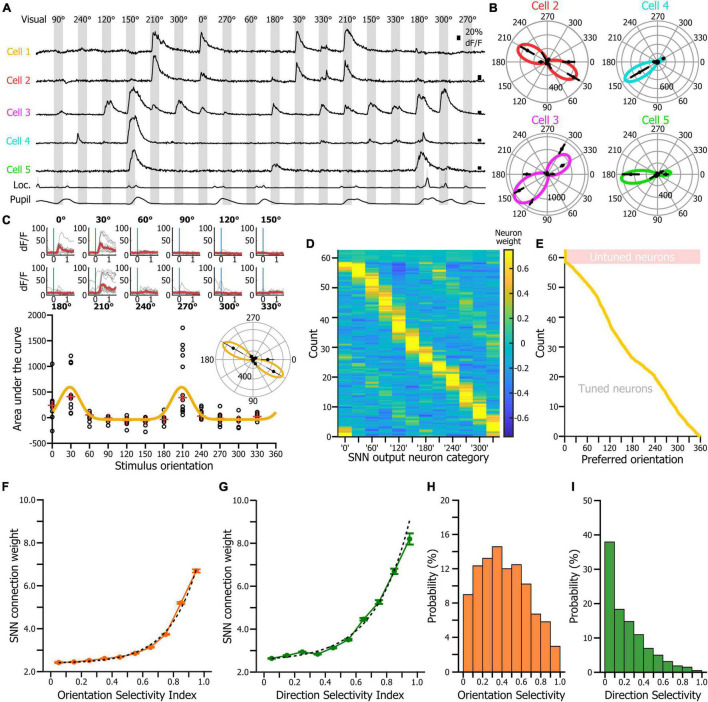
SNN connection weights as a function of the preferred orientation, orientation selectivity and direction selectivity of the input neurons. **(A)** Activity of the neurons labeled in Figure 1D during an 18-trial segment of the tuning block. **(B)** Tuning curves of the neurons 2 to 5 shown in panel **(A)**. Radial axis: area under the curve of the neuronal response. **(C)** Tuning curve of the cell 1 shown in panel **(A)**. Top: superimposition of the fractional fluorescence of the neuron for all the trials of the different stimuli of the tuning block. In red is the average of the fractional fluorescence across trials. Bottom: tuning curve (orange line) fitted to the data points (dots). Red crosses indicate the median value across trials. Inset: same tuning curve presented as a polar plot. **(D)** Connection weights (see color scale) of each input cortical neuron for the 12 SNN outputs (*x*-axis). The neurons were ranked by their preferred orientation (*y*-axis). Connection weights were normalized using a *z*-score normalization method. **(E)** Distribution of the preferred orientations in the input cortical neurons’ population. Presentation matching the presentation shown in panel **(D)**. **(F)** Distribution of the input neurons’ connection weights as a function of the V1 neurons’ orientation selectivity indexes. Dotted lines: exponential fit. **(G)** Distribution of the input neurons’ connection weights as a function of the V1 neurons’ orientation selectivity indexes. **(H)** Distribution of the orientation selectivity index in the recorded neuronal population. **(I)** Distribution of the direction selectivity index in the recorded neuronal population.

In an effort of comparison, our first goal was to assess whether the SNN would use the same features of the neurons’ response statistics that are captured by the traditional tuning curves. Therefore, we tested the hypothesis that the weight of the cortical neurons in the SNN decision corresponded to their orientation tuning properties. In a neural network, the relative contributions of the input variables to the predictive output depend primarily on the magnitude and direction of the connection weights between computational neurons. Input variables with larger connection weights represent greater intensities of signal transfer (Olden and Jackson, 2002). Therefore, they are more important in the prediction process compared to variables with smaller weights. To determine whether the orientation tuning of the cortical neurons would be a predictor of their connection weight in the SNN, we first determined the preferred orientation, orientation selectivity index (OSI) and direction selectivity index (DSI) for all the neurons of the dataset. For each neuron, the responses to the different visual stimuli of the tuning block (Figure 3A) were fitted using a resampling-based Bayesian method (Cronin et al., 2010; McClure and Polack, 2019; Figures 3B,C). We then estimated the weights of every input cortical neuron for each of the 12 SNN outputs (corresponding to the 12 visual stimuli of the tuning block) using the *Connection Weight Approach* (Olden and Jackson, 2002; Olden et al., 2004), and repeated this measurement in 250 SNNs (250 inputs × 250 iterations = 62,500 datapoints). Finally, we sorted the cortical neurons by preferred orientation and displayed their connection weights for each of the 12 decision outputs (Figure 3D). We found that the SNNs assigned the largest connection weights to neurons tuned to the visual stimulus presented (Figures 3D,E). We then plotted the connection weights of cortical neurons as a function of their orientation selectivity (Figure 3F) and direction selectivity indexes (Figure 3G). Those two relationships were best fitted by an exponential curve indicating that cortical neurons with high orientation and/or direction selectivity had a much larger connection weights, and therefore a much larger impact in the SNN decision than most of the other cortical neurons, even though they represented only a fraction of the total neuronal pseudo-population in V1 (Figures 3H,I). Hence, we show that SNNs classify the orientation of the visual stimuli of the tuning block by learning and using the orientation tuning properties of the V1 neurons.

### Sound Modulation of Orientation Representation in Naïve Mice V1

**FIGURE 4:**
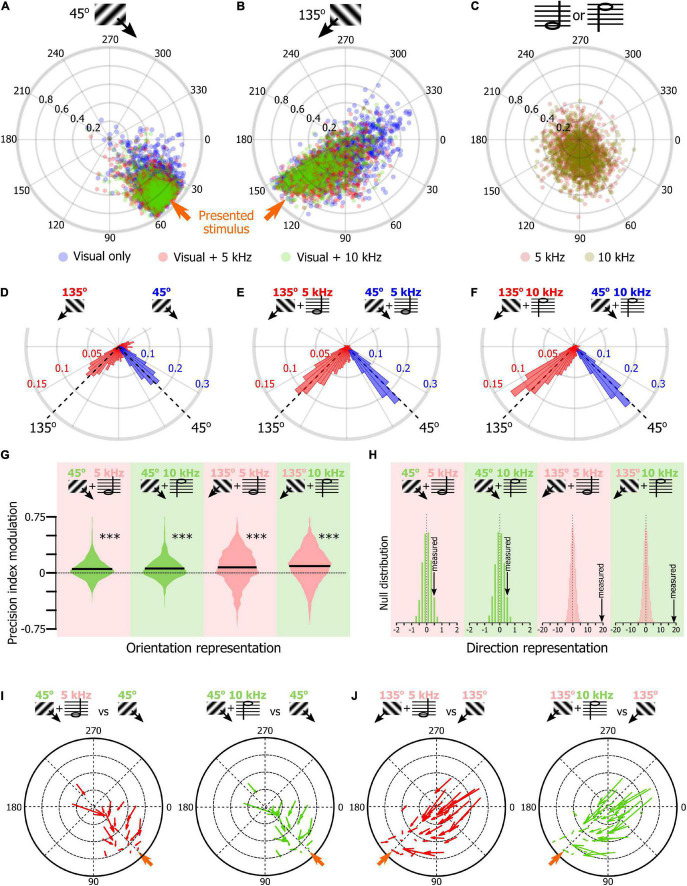
Sound modulation of the V1 population evoked response in naïve mice. **(A)** Output of 1,000 SNNs made of 250 randomly selected V1 neurons to the presentation of a 45° drifting grating in the unimodal (blue) and audiovisual (5 kHz tone: red; 10 kHz: green) contexts. The orange arrow indicates the orientation of the presented stimulus. **(B)** Same representation as in panel **(A)** for the presentation of the 135° drifting grating. **(C)** Same representation as in panel **(A)** for the presentation of unimodal auditory stimuli (5 kHz tone, dark red; 10 kHz, dark green). **(D)** Distribution of the orientations indicated by the output circular mean of the 1,000 SNNs shown in panels **(A,B)** when the input is the neuronal activity evoked by the unimodal 45° (blue) and unimodal 135° (red) drifting grating. **(E)** Same representation as in panel **(D)** when the visual stimulus is paired with the 5 kHz tone. **(F)** Same representation as in panel **(D)** when the visual stimulus is paired with the 10 kHz tone. **(G)** Modulation by the 5 kHz (red background) and 10 kHz (green background) sounds of the precision indexes of the 1,000 SNN shown in panels (**A,B)**. The black bar indicates the mean of the distribution. ^***^ Random permutation test (*p* < 0.0001). **(H)** Comparison of the percent of SNN outputs shown in panels **(A,B)** changing direction when the visual stimulus was presented with one of the two sounds in panels **(B,C)** with the probability distribution of the same measure performed 10,000 times with shuffled data (from left to right: *p* = 0.02, *p* = 0.02, *p* < 0.0001, *p* < 0.0001). **(I)** Quiver plot of topographically clustered modulation vectors illustrating how SNNs with similar outputs for the presentation of the 45° drifting grating are modulated by the 5 kHz (red) and 10 kHz (green) sounds. **(J)** Same representation as in **(I)** for the presentation of the 135° drifting grating.

Once we had confirmed that SNNs were using the orientation tuning properties of the V1 neurons to classify the visual stimuli of the tuning block, we tested the hypothesis that the SNN approach could be used to determine how sound modulates the representation of the orientation representation in V1 L2/3. We trained 1,000 SNNs to classify the stimuli of the tuning block. Then, we presented the SNNs with the response of their input cortical neurons to the presentation of the 45° drifting gratings recorded during the unimodal visual block (average output of 100 resampled trials). The circular means of the 1,000 SNNs outputs were displayed on a polar plot (Figure 4A, blue dots, see Figures 2E,F for the approach). We repeated the same analysis for the 45° drifting gratings recorded during the audiovisual blocks when the visual stimulus was paired with the low tone (5 kHz, red) or the high tone (10 kHz, green). The same approach was used with the neuronal response to the presentation of the 135° drifting gratings (Figure 4B; unimodal: blue, audiovisual 5 kHz: red, audiovisual 10 kHz, blue) and for the unimodal auditory tones (Figure 4C). In the unimodal visual and audiovisual conditions, the output vectors of the SNNs were indicating the orientation of the visual stimulus (Figures 4D–F; circular mean ± confidence interval; For 45°: unimodal visual: 42.3° ± 1.0, visual + 5 kHz tone: 47.7° ± 0.8, visual + 10 kHz tone: 48.0° ± 0.7; For 135°: unimodal visual: 105.4° ± 4.4, visual + 5 kHz tone: 123.0° ± 2.7, visual + 10 kHz tone: 127.9° ± 2.7), while in the unimodal auditory condition the output vectors were both similarly attracted toward 90°, i.e., at equidistance between 45° and 135° (5 kHz tone: 84.6° ± 5.0, 5 kHz tone: 89.0° ± 4.3; Figure 4C). To determine how sounds modified the representation of the visual stimuli in V1, we compared the accuracy of the representation of the visual stimulus in the unimodal and audiovisual conditions by computing for each SNN the difference between the precision index (see Figure 2F) obtained with the audiovisual responses and the precision index obtained with the unimodal responses. We then plotted the distribution of those differences as violin plots (Figure 4G). We found that the precision index of the representation of the 45° and 135° stimuli was improved in the audiovisual conditions compared to the unimodal context (Figure 4G; difference between 45° + 5 kHz and 45° unimodal; 5.4%; 45° + 10 kHz and 45° unimodal; 6.1%; 135° + 5 kHz and 135° unimodal; 7.6%; 135° + 10 kHz and 135° unimodal; 9.4%; *p* < 0.0001 for all audiovisual combinations; random permutation test). We also compared the proportion of SNN outputs that changed direction (e.g., moving from a 225° output to 45°) when the visual stimulus was presented with one of the two sounds. We found that the representation of the stimulus direction was more accurate when the stimulus was presented with a tone (Figure 4H; random permutation; *p* = 0.02, and *p* < 0.0001, respectively for 45° and 135°combined with either tone). This improvement of the representation of the 45° and 135° visual stimuli was mainly due to the improvement of the SNNs that performed the worst, as illustrated by the quiver plots indicating how the SNNs outputs were modified by sound as a function of the output of the SNN for the unimodal stimulus (Figures 4I,J, the base of the arrow corresponds to the unimodal stimulus while the arrowhead corresponds to the audiovisual stimulus). Altogether, those results replicated our previous findings that orientation is better represented in the V1 of naïve mice when sounds are presented simultaneously with the oriented visual stimulus (McClure and Polack, 2019).

### Sound Modulation of Orientation Representation in Mice Performing an Audiovisual Task

**FIGURE 5:**
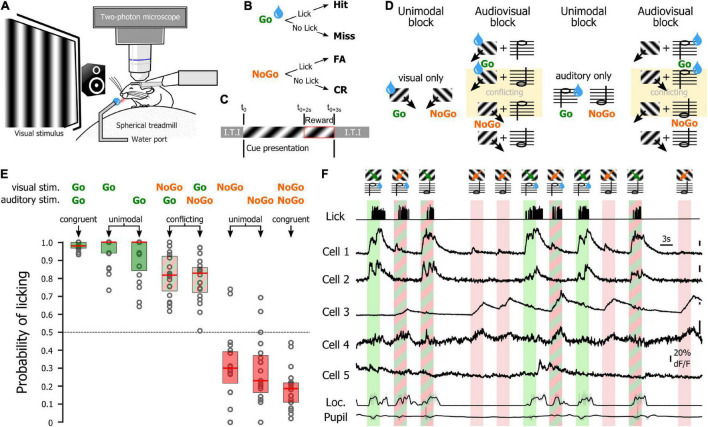
Recording sessions in trained mice. **(A)** Schematic representation of the recording setup. **(B)** Potential behavioral outcomes for a trial. **(C)** Trial time course. Inter Trial interval (I.T.I) after Hit and CR: 3 s; I.T.I after Miss and FA: 12.5 s. **(D)** Stimuli shown during the unimodal, audiovisual, and tuning blocks in a session starting with visual reward. Water drops indicate stimuli that are rewarded for that block. **(E)** Mouse licking probability for the different unimodal and audiovisual stimuli. **(F)** Activity of neurons during the presentation of the visual stimuli of the audiovisual block (auditory reward). The neuronal activity was recorded simultaneously with the licking activity (top trace), the locomotion of the animal as well as the pupil size (bottom traces).

We then tested the hypothesis that the modulation of the representation of the orientation of visual stimuli by sounds depended on the relative importance of the auditory and visual stimuli for the completion of the task. To test this hypothesis, we used a new database in which mice were performing an audiovisual discrimination task using the same stimuli as the one presented to the naïve mice. Water-restricted mice were placed on the same apparatus as the naïve mice, but this time a lickometer was placed in front of their mouths (Figure 5A). Mice were successfully trained at performing the unimodal visual and the unimodal auditory Go/NoGo task (Figure 5B, the training order was randomly assigned). For those two tasks, mice were presented with a Go cue (for the visual task a 45° drifting grating; for the auditory task a 10 kHz tone) or a NoGo cue (visual task: 135°; auditory task: 5 kHz tone). The stimulus was presented for 3 s. Mice had to lick to obtain a reward when the Go signal was presented, and to withhold licking during the NoGo signal. The response window corresponded to the third second of the stimulus (Figure 5C). Once trained to the first unimodal task, mice were trained to the other unimodal task. Then, when the expert level (*D*′ > 1.7) at those two unimodal tasks was reached, mice were habituated to the audiovisual context (Figure 5C). Each session of the audiovisual task started with a unimodal block (either visual or auditory) followed by an audiovisual block during which the modality of the preceding unimodal block was predicting the reward (Figure 5D). This first audiovisual block was followed by a unimodal block using the other modality (either auditory or visual, respectively) then a second audiovisual block during which the modality of the second unimodal block was used to dispense the reward. To perform perfectly at the task, mice would have to perform a modality-specific attention task (attend visual for the first two blocks then auditory for the last two blocks in the example provided in Figure 5D). Our analysis of the mouse behavior showed that mice used an alternate strategy (Figure 5E). Indeed, they licked whenever one of the Go cues (auditory or visual) was presented, regardless of the identity of the rewarded modality for the current block (auditory rewarded block lick rate (median ± m.a.d.): Go_*v*_-NoGo_*a*_: 71 ± 12%; NoGo_*v*_-Go_*a*_: 92 ± 5%; visual rewarded block lick rate: Go_*v*_-NoGo_*a*_: 94 ± 5%; NoGo_*v*_-Go_*a*_: 68 ± 20%). Therefore, we sorted the data by presented stimuli, pooling together audiovisual blocks where different modalities were rewarded. In the unimodal condition, mice licked systematically whenever the Go signal was presented (unimodal auditory hit rate: 92%, *n* = 10 mice, Wilcoxon test: *p* < 0.0001; unimodal visual hit rate: 96%, *n* = 10 mice; Wilcoxon test: *p* < 0.0001), and avoided licking in the presence of the NoGo signal (unimodal auditory False Alarm (FA) rate: 29%, *n* = 10 mice, Wilcoxon test: *p* < 0.0001; unimodal visual FA rate: 32%, *n* = 10 mice, Wilcoxon test: *p* = 0.0004). In the audiovisual blocks, our analysis of the mouse behavior showed that mice used an alternative strategy. The performance of the mice at refraining from licking was improved when the auditory and visual NoGo cues were presented simultaneously, compared to the unimodal NoGo conditions (audiovisual NoGo FA rate: 19%, *n* = 10 mice, audiovisual NoGo vs. unimodal auditory NoGo: Wilcoxon test *p* = 0.0100; audiovisual NoGo vs. unimodal visual NoGo: Wilcoxon test *p* = 0.0009). We did not find an improvement in behavioral performance when the two Go signals were presented together, compared to the two unimodal conditions (hit rate 98%, *n* = 10 mice; audiovisual Go vs. unimodal auditory Go: Wilcoxon test *p* = 0.8109; audiovisual Go vs. unimodal visual Go: Wilcoxon test *p* = 0.2415), likely because mice already performed almost perfectly in the unimodal contexts. When the visual and auditory-visual cues were in conflict, mice clearly chose to lick (Go_*visual*_/NoGo_*auditory*_: hit rate = 79%, *n* = 10 mice; Wilcoxon test: *p* < 0.0001; NoGo_*auditory*_/Go_*visual*_: hit rate = 82%, *n* = 10 mice; Wilcoxon test: *p* < 0.0001). Hence, when the signal was conflicting (e.g., Go visual paired with NoGo auditory), mice licked by default (Figure 5E). The apparent strategy of the animals was to seek the Go cue regardless of the modality, ignoring the current reward contingencies. Instead of the intended modality-specific attention Go/NoGo task, they engaged with the task as a cross-modal Go detection task (Figure 5F).

**FIGURE 6:**
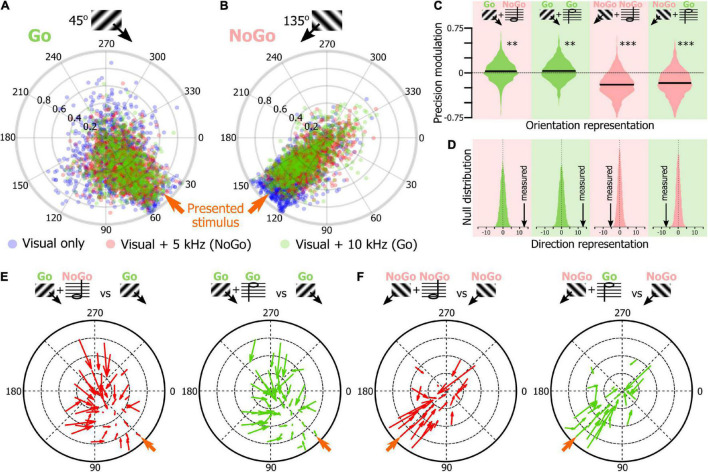
Sound modulation of the V1 population evoked response in mice performing an audiovisual detection task. **(A)** Output of 1,000 SNNs made of 250 randomly selected V1 neurons to the presentation of a 45° drifting grating (Go cue) in the unimodal (blue) and audiovisual (5 kHz tone: red, NoGo cue; 10 kHz: green, Go cue) contexts. The orange arrow indicates the orientation of the presented stimulus. **(B)** Same representation as in panel **(A)** for the presentation of the 135° drifting grating (NoGo cue). **(C)** Modulation by the 5 kHz (red background, NoGo cue) and 10 kHz (green background, Go cue) sounds of the precision indexes of the 1,000 SNNs shown in panels **(A,B)**. The black bar indicates the mean of the distribution. Random permutation test (^**^p = 0.002; ^***^p < 0.0001). **(D)** Comparison of the proportion of SNN outputs shown in panels **(A,B)** changing direction when the visual stimulus was presented with one of the two sounds in panels **(B,C)** together with the probability distribution of the same measure performed 10,000 times with shuffled data (for all panels: *p* < 0.0001). **(E)** Quiver plot of topographically clustered modulation vectors illustrating how SNNs with similar outputs for the presentation of the 45° drifting grating (Go cue) are modulated by the 5 kHz (red, NoGo) and 10 kHz (green, Go) sounds. **(F)** Same representation as in panel **(E)** for the presentation of the 135° drifting grating (NoGo cue).

Using the SNN approach, we compared the representation of the Go and NoGo visual cues in the unimodal and audiovisual contexts (Figures 6A,B). The orientation of the SNNs output vectors were similar for the unimodal and audiovisual blocks (circular mean ± confidence interval; For 45°: unimodal visual: 61.7° ± 4.3, visual + 5 kHz tone: 58.7° ± 2.7, visual + 10 kHz tone: 57.8° ± 2.8; Figure 6A; For 135°: unimodal visual: 130.5° ± 2.3, visual + 5 kHz tone: 128.7° ± 2.9, visual + 10 kHz tone: 133.1° ± 3.1; Figure 6B). The precision of the representation of the visual Go signal (see Figure 2F) was slightly but significantly improved by sound (Figure 6C; difference between 45° + 5 kHz and 45° unimodal; 2.9%, *p* = 0.003; 45° + 10 kHz and 45° unimodal; 3.2%; *p* = 0.002, random permutation test). On the contrary, the representation of the NoGo signal was significantly less precise in the audiovisual context (Figure 6C, 135° + 5 kHz and 135° unimodal; −19.8%, *p* < 0.0001; 135° + 10 kHz and 135° unimodal; −17.1%; *p* < 0.0001; random permutation test). This opposite modulation of the Go and NoGo orientation representation was associated with a comparable change of the representation of the direction of the drifting grating with a significant improvement of the representation of the direction of the Go drifting grating in the audiovisual context, and a deterioration of the representation of the direction of the NoGo drifting grating with sound (Figure 6D). The differential modulation of the Go and NoGo cue representation by sound was particularly salient in quiver plots as most of the improvement of the Go cue representation was carried by SNNs having a poor accuracy in the unimodal context (Figure 6E), while most of the modulation for the NoGo visual cue representation was due to highly accurate SNNs that saw their performance decrease in the audiovisual context (Figure 6F). Altogether our results suggest that sounds can have a bidirectional impact on the orientation representation accuracy in V1, as the modulation interacted with the way the animals engaged in the task. For the sought-after Go visual stimulus, sound potentiated the orientation representation, while it degraded the representation of the NoGo visual stimulus that the animals tended to ignore.

## Discussion

In this study, our goal was to test the SNN approach as a tool to analyze the representation of the orientation of drifting gratings by the V1 neuronal population. As an example, and to provide a comparison with a more traditional analysis approach, we used this method on a previously published dataset investigating the modulation by sounds of orientation representation (McClure and Polack, 2019). We showed that: (1) SNNs with a unique hidden layer of 10 computational neurons can be trained to categorize the 12 orientations of the tuning block. (2) For each output node (corresponding to the different orientations of the tuning block), SNNs assign to each input node a connection weight that corresponds to the tuning of the input cortical neuron for that orientation. (3) The circular mean of the SNN output can be used to estimate the orientation of drifting gratings even when the SNN was not trained to categorize that orientation. (4) Using this approach, we confirmed that orientation representation is improved in naïve mice when a sound is presented simultaneously with the visual stimulus. (5) Finally, we extended the approach to a new dataset and showed that in mice performing a cross-modal Go detection task the sound-induced modulation of the V1 orientation representation depends on the importance of the visual stimulus for the behavior. Indeed, sounds improved the representation of visual stimuli that acquired a high behavioral importance for the animals (the Go signal) while degrading the representation of the other visual stimuli (the NoGo signal).

This study is, to our knowledge, the first study using SNNs to evaluate the representation by the V1 neuronal population of the orientation of visual stimuli. The use of SNNs to analyze the structure of neuronal activity of the visual cortex brings convolutional networks back to their roots, as its original structure was inspired by the connectivity of the vertebrate visual system (Fukushima, 1980). Recently, convolutional networks started to be used to model sensory processing in V1 and were found to be more effective than other traditional methods (Zhang et al., 2019). In our study, the SNNs received the input of a subset of V1 L2/3 neurons and were trained to categorize their activity patterns evoked by the stimuli of the tuning block. Their high accuracy for classifying the trained input patterns quickly led us to abandon the idea of training SNNs to discriminate between the 45° and 135° visual stimuli of the unimodal block. Indeed, this approach leaves very little room for improvement as the classification of the trained stimuli is highly efficient. Moreover, training the SNN to classify the unimodal block stimuli would not be addressing the question of orientation representation, but estimate the capability of the SNN to use the neuronal activity to discriminate between the two stimuli; an approach similar to that of studies using linear discriminant analysis (Stringer et al., 2021). Thus, we devised the alternate strategy of using the output of SNNs trained to categorize the V1 neuronal activity evoked by the 12 orientations of the tuning block, allowing us to assess how the orientations of the unimodal and audiovisual stimuli were represented in V1. Indeed, as the output layer of the SNNs uses a softmax function, SNN outputs indicate the probability that the presented visual stimulus belongs to the different trained stimulus orientation categories. By linearizing the categorical outputs in the orientation space using the circular mean, we were able not only to estimate the orientation of the visual stimulus, but also the specificity of this neuronal pattern.

For this report, we chose to train hundreds of SNNs with different subsets of the cortical neurons present in our databases. Our goal in using this approach was to assess the statistical variability of the orientation representation across the V1 neuronal population. We found large variations in SNN performance depending on the subset of neurons used. The poor performance of some SNNs is likely due to samples with a small proportion of well-responsive neurons. This would explain why the presence of sounds is particularly efficient at improving the performance of SNNs poorly responding in the unimodal context (as shown in the quiver plot analysis Figures 4I,J). Indeed, we had already shown that the improved representation of oriented stimuli in the audiovisual context is due to an increase in the response of tuned neurons and a decrease in response of neurons not tuned for the stimulus (McClure and Polack, 2019), limiting the risks of misclassification due to outlying neuronal activities. The main advantage of the SNN approach compared to the approach used in our previous study (McClure and Polack, 2019), is that all the neurons are now included in the analysis. During training, every computational input neuron is given a connection weight proportional to the importance of this neuron in the assessment of the classifier output (Garson, 1991; Goh, 1995). The possibility of determining the connection weights using the *Connection Weight Approach* (Olden and Jackson, 2002; Olden et al., 2004) is a great strength of SNNs. Hence, we show that for each categorical output (i.e., the 12 orientations of the tuning block) the largest weights are attributed to neurons having similar preferred orientations. Moreover, we show that neurons with the largest weight are neurons with the best orientation selectivity and/or best direction selectivity. Thanks to the *Connection Weight Approach*, it will be possible in future studies to determine which neuronal population drives the sound modulation of orientation representation, and whether it happens through mechanisms such as potentiation of tuned neurons, suppression of untuned neurons, or improved trial to trial reliability of the neurons.

We used the SNN approach on two databases that were generated to investigate how sound modulates the visually evoked neuronal activities in V1. Indeed, in the past decade, an increasing number of studies have shown that the presence of sounds modifies the response of neurons to the presentation of visual stimuli in the mouse V1 (Iurilli et al., 2012; Ibrahim et al., 2016; Meijer et al., 2017; Deneux et al., 2019; Knöpfel et al., 2019; McClure and Polack, 2019; Garner and Keller, 2021). Those studies are characterized by a large array of recording techniques (electrophysiology and functional imaging), different sounds and visual stimuli, and a great variety of analysis approaches. The database of naïve mice used in this study was generated for our previous report in which we showed that the presence of pure tones improves the representation of the orientation and direction of the visual stimulus in V1 L2/3 by favoring the recruitment of a neuronal population better tuned to the visual stimulus orientation and direction than the population responding to the unimodal visual stimulus (McClure and Polack, 2019). Here, we confirmed using the SNN approach that the presence of pure tones improves the representation of the orientation of the visual stimuli. This new approach allows us to assess orientation representation by the whole population and not using a subset of selected “active” or “responsive” neurons (Ibrahim et al., 2016; Meijer et al., 2017; Deneux et al., 2019; McClure and Polack, 2019). We also confirmed that sound modulation is stronger in V1 neurons that poorly respond to the visual stimulus in the unimodal context, or that are biased toward the opposite direction (Figures 4I,J; McClure and Polack, 2019). Note that we had already shown that arousal and locomotion could not account for those results (McClure and Polack, 2019), and we therefore did not consider those parameters further in this study. Moreover, several studies have recently demonstrated that the modulation of the V1 neuronal activity by behavioral parameters such as locomotion and arousal are orthogonal to orientation encoding (Hajnal et al., 2021; Stringer et al., 2019).

We also present novel findings suggesting that sound modulation itself depends on the audiovisual context. Indeed, we show in mice performing a cross-modal Go detection task that the presence of sounds improves the representation of the Go visual cue orientation while degrading the representation of the NoGo visual cue orientation. This degradation of the NoGo visual cue orientation representation is mostly carried by the degradation of the best performing SNNs. This suggests that this effect is supported by a decrease in the responsiveness of highly tuned neurons. This result extends previous findings showing that incongruent audiovisual stimulation (a looming visual stimulus associated to a frequency-modulated tone) had a suppressive effect on V1 neuronal responses while congruent audiovisual stimuli did not significantly change the neuronal responses in V1 [(Meijer et al., 2017) but see also (Garner and Keller, 2021)]. In our behavioral paradigm, we did not find an effect of behavioral congruence as both NoGo and Go sounds similarly suppressed the NoGo visual cue while improving the Go visual cue orientation representation. Future experiments will be necessary to determine the cellular and network mechanisms underpinning the differential modulation of V1 visual processing by sound.

## Data Availability Statement

The raw data supporting the conclusions of this article will be made available by the authors, without undue reservation.

## Ethics Statement

The animal study was reviewed and approved by the Institutional Animal Care and Use Committee (IACUC) of Rutgers University–Newark.

## Author Contributions

JM and P-OP designed the project. JM performed the experiments. P-OP performed the analyses with assistance from OBE, JC, and JM. P-OP wrote the manuscript with assistance from JC. All the authors revised the manuscript.

## Conflict of Interest

The authors declare that the research was conducted in the absence of any commercial or financial relationships that could be construed as a potential conflict of interest.

## Publisher’s Note

All claims expressed in this article are solely those of the authors and do not necessarily represent those of their affiliated organizations, or those of the publisher, the editors and the reviewers. Any product that may be evaluated in this article, or claim that may be made by its manufacturer, is not guaranteed or endorsed by the publisher.
